# Necropolitics and Diffuse Violence: Critical Reflections on Social Discourses About the LGBT Body

**DOI:** 10.3389/fsoc.2021.633975

**Published:** 2021-06-18

**Authors:** Cristine Jaques Ribeiro, Camila de Freitas Moraes, Lívio Silva de Oliveira

**Affiliations:** ^1^ Universidade Católica de Pelotas, Pelotas, Brazil; ^2^ Federal University of Rio Grande do Sul, Porto Alegre, Brazil

**Keywords:** city, necropolitics, diffuse violence, LGBT body, resistance

## Abstract

This article aims to discuss the state’s function and the speeches about the LGBT body from the theoretical concept of necropolitics, defended by Achille Mbembe, which apprehends that this is a sovereign state that subdues, oppresses, and acts for the management of politics of death and applies them on bodies and populations, determining who is subject to live or die. The concept of necropolitics will be enunciated with diffuse violence as a systemic concept that demarcates social relations, and forms of sociability in contemporaneity as defensive behaviors that can legitimize human rights violations. Diffuse violence recognizes the increasing criminal rates, especially homicides and patrimonial offenses, as the main factor in producing diffuse fear, connoting a generalization of the feeling of insecurity. However, this dynamic also points out which types and social groups are most vulnerable to violence. Based on these theoretical and methodological contributions, we will seek to understand how social discourses and their implications are transversal to the LGBT body and how they manifest through oppression.

## Introduction

The cities are a social and historical phenomenon verified on all continents. These materially constructed spaces throughout human existence have represented a transformation of sociability and civility. The circulation of goods, people, and ideas has constituted the symbolic and historical aspects of cities, which varied from place to place. They indicate cultural, economic, and legal features that form social cohesion, which unfolds in certain collective norms, values, and rules. In this sense, these “order” dynamics, specific or universalized, demonstrate the asymmetric power relations *in* and *of* the city.

We identified that these urban asymmetries have functional, material, and symbolic characteristics, and they determine the territorial segmentation of cities. The functional and material aspects can be understood as directly connected, indicating the social division of labor in the urban space. In these aspects, we observe the infrastructure for the circulation of goods and people for leisure, production, consumption, among other actions, as well as housing and territorial qualification issues. The symbolic aspects produce the sense and the meaning of the territories, understanding the territory as a category that is not restricted to the geographical dimension but that can also denote affective, social, and historical aspects. Thereby, these articulated dimensions produce and categorize individuals, collective identities, and subjectivities, which can catalyze social conflicts.

At first, the city is characterized as a place of conflict. The proposal for urban universality perhaps can be circumscribed in an ontological conflict with the notion of plurality, considering that universality suggests a hegemonic thinking, that is, an ideal way that emerges as real to a certain extent, while plurality admits the possibility of conflict but also the coexistence of distinct and even antagonistic social groups. When we take subjection as a variable for territorial segmentation of cities, we note the modes of population administration and management, alluding to forms of social control based on behavioral issues and diffuse components, such as race, class, and gender. Thus, these dynamics compose the types of existence in the city and define who is a “*citizen*” from a homogeneous and universalized standard, which can marginalize social groups that do not fit in it.

Based on these foregoing arguments, the present article aims to analyze the social control policies of these marginalized social types. Our unit of analysis is the LGBT body as a dissident body. In this sense, our theoretical premise is the social policies applied to these historical individuals, based on the articulation between the theoretical concepts of necropolitics and diffuse violence. Thus, our intention was to understand the forms of oppression and the mechanics and strategies of claiming the LGBT population in the urban space.

## Who Does Exist in the City?

When dealing with the city theme, we are considering that it does not fit into a universal conceptualization. Considering the conceptual complexity, it is important, at this moment, to problematize how urban planning welcomes or does not welcome its “city dwellers” (Agier, 2011). During the different historical configurations, we verify the implementation of ordered rationality about the organization of the public space that advanced in the technical and technological dimension and disregarded the existence of heterogeneous lifestyles. This ordered reality shapes a city without residents, that is, a city geared toward the desires of the global real estate complex and away from the local reality that pulsates in the territories of existence.

So, the central question is Who exists in the city? This question invites us to problematize what city we are talking about and to whom this city is directed. Therefore, it is first a matter of verifying the advancing processes of financialization of urban territory, which is presupposed to be the financialization mechanism of the bodies of the city. There is a type of city management that legitimizes the use of the public space as private property, and this form of management “*is shared between the State and the private sector*” (Tietboehl, 2015:26). Thus, the construction of the city takes place without people—what matters are the financial investments destined to produce a city as a product for speculation.

In the book *Direito à cidade* ^1^ (the right to the city) by Henri Lefebvre (2011), it is possible to comprehend the dimension of defending the ways of existing in the urban space as the right to inhabit in the city and therefore as a “right to urban life”^2^ (Lefebvre, 2011: 118). However, the theme of urban space evidences that there is a distance between the urban planning of cities and the assuring of quality of urban life for their inhabitants. Why does this distance exists? The rationality of planning is strengthened in the perspective of control from the regulation of the use of public spaces, which aims to make the city more favorable for the private sector. As Maricato argues, “*the disputes over the appropriation of real estate rents determine, to a large extent, the fate of cities and their development*”^3^ (Maricato, 2013: 83).

From the previous statements, it is possible to question whether there is a city or there are multiple cities in the same urban space as it is also possible to think about reinventing another nomenclature that could comprehend the heterogeneous modes of existence denied by public administrations and their planners. However, the problematic that is established has its origins in the defense of private property, in which the agrarian issue is central to understanding that the current city exists for those who are property owners. Cities are the result of an intense historical process of concentration of land in rural areas. Since the promulgation of land law in Brazil, in 1850 (Silva, 1999), we will legally experience the denial of the right to exist for the sake of the right to property. According to Foucault, “the law does not originate from nature […] the law rises from burned cities, from devastated lands; it rises with the famous innocents in agony while the day dawns” (Foucault, 2018: 43).

As Raquel Rolnik affirms about Brazil’s Statute of the City, despite the promises of decentralization and extension of the right to the city included in the document, the signs of the predatory and discriminatory model of the city remain in full force (Rolnik, 2015: 266). Therefore, returning to the initial question about who exists in the city, we realize that the city is driven by strategic technicality and existence is negotiable or disposable.

“*The plans produce norms destined to not be carried out, thus creating an abyss between the* ‘*real city*’ *and the* ‘*legal city*’”^4^ (Ribeiro and Cardoso, 1996: 65). This reality is the result of the colonization constituted in the use of urban land, in the denial of the social function of the land, and therefore in the right to property at the expense of the right to life. In this sense, Milton Santos argues that global money is a hard and relentless tyrant, never seen before in this intensity throughout the history of humanity. (Santos et al., 2011: 17).

So, the existence in or of the city depends on the regulation imposed by urban planning, through the control mechanisms of society. In other words, this dynamic presents rationality based on the imposition of marking bodies of “errant” heterogeneity. But, who are these heterogeneous bodies? For instance, they are the ones who suffer from segregation processes or from some kind of “normalization” for social “coexistence.” These institutionalized codes in social relations are the result of the patriarchy and structural racism that have been perpetuated in the current system. Thus, the evidence of these codes is established in public speeches, socially produced and shared (by media and Internet, e.g.), that reinforce stigmas and that can fuel violent practices. Those discursive and interventional practices are strengthened by the social imaginary about what sexuality is and how it should be treated in the “planned” city.

## The Death Policy on Dissident Bodies and Sexualities

The binary difference about sexuality and bodies serves to determine the issues concerning the reproduction of the species, as well as a regime of sexualities and their modes of enjoyment. This includes, above all, the universalization of heterosexuality as a social and reference model for any and all forms of sexual diversity.

Therefore, to stand before sexuality understood as dissident, sinful and perverted by moralizing, religious, and cultural speeches is to understand that discursive action has power over the body of another. Those speeches that launch into the social, political, economic, and scientific techniques of knowledge are a contiguous attempt to discipline, heal and/or regulate behaviors, bodies, and thoughts considered antagonistic to the imposed norms, being a power system that regulates practices (Foucault, 1987: 9). In this perspective, Foucault led us to infer that it is through the discourse that all individuals produce themselves materially and there is no escape from this premise. The discursive formations influence the social practices of individuals inserted in power relations, in order to produce hegemonic positions in which both individuals and institutions act.

Foucault (2013) argues that the discourse takes place in a set of invisible practices in the economic, political, linguistic, and social spheres, for example, but always in a given temporal space. In other words, in the enunciative function of each period, the discourse can remain or fade away. The speeches understood as practices of a given power can operate and be perceived as continuous or discontinuous techniques, which can intersect at times or be ignored or eliminated (Foucault, 2013). Therefore, when we think about the foundation of modern society, it was made on clerical, political, and economic concepts about the universalization of bodies, pleasures, and ways of existing that are based on the perspective of white, elitist, and heterosexual men. From this framework, “truths” are constructed about sex, gender, ethnicity, and sexuality, as well as about those bodies whose differentiation have always occurred with the ultimate intention of maintaining hegemonic interests, whether of the state or of institutions such as schools, universities, and hospitals.

Foucault reaffirms that the discourse production selects, controls, and dominates its random event, evading its heavy and fearsome materiality (Foucault, 2013: 9). It means that the mark of difference aligns with indifference, exclusion, and maintenance of stigmatizing discourses. It is not just about bodies demarcated by difference or diversity but about the stratification of ways of existing. Thus, LGBT bodies, for example, are distinct by apathy and by material and subjective death because they are considered to have transcended sexual binarism.

However, Foucault’s thinking based on discourse analysis and the concepts of biopower and biopolitics teaches us, *a priori*, that discourses emanate from power relations to exclude or include a given individual in the social field, as well as to construct truths about them. In another point, the policy mentioned by Foucault is presented as a possibility of life regulation and population control, where bodies are regulated by the state when there is an implementation of an anatomy policy of the body.

In other words, the former discursive action is put into effect in the materiality of the entire population that is now presented in a dyad: incorporation versus disincorporation. More than launching universalizing individuals, biopolitics assembles, in the same logic, individuals who maintain relationships, behaviors, sexualities, and ways of living that are common among themselves. The public body is put on display to be regulated by a sovereign state, whose power lies in deciding which lives are liable to live or die. Life extension is made feasible by medical knowledge, through practices that allow the managing of birth rates, the detecting and controlling of epidemics, the increase in longevity, among others. On the other end of the spectrum, there are punishments for bodies and individuals that violate legal norms, rules that are enforced by incarcerations. The state also has hold in the discipline of time and the sustaining of an economic extrapolation of the working class, as well as in the enrichment of those who have economic power. All of the above are examples of how biopolitics develop (Foucault, 1999).

Thinking about the colonized and usurped territories by the image of the European colonizer, the philosopher Achille Mbembe (2011) goes back to a Foucaultian notion to point out that the policy on bodies where there are marks of difference and disincorporation is not regulated by biopolitics, but by necropolitics. Mbembe says that the sovereign state and the entire institution that derives from it, more than universalizing bodies, seek to disincorporate them, erase their differences and their singularities through the bias of death (material, subjective and institutional). For this, the philosopher uses colonial historicity to explain how these processes occur in the enslaved body; the same logic can be used when analyzing the LGBT body.

Mbembe (2018) notes that there are bodies in contemporary times that suffer from hostility and persecution. The state advocates death zones, social disparities, and the annihilation of the bodies of LGBT, black people, women, and other minorities through the systemic and structural violence presented by LGBTphobia, racism, sexism, and other forms of oppression that have taken root since the colonial process. The operationalized violence in the colonized lands is resignified in modern societies through thoughts, attitudes, and behaviors that are intertwined with the state apparatus in order to exterminate any individuals who escape the hegemonic principles of social organization, especially the LGBT bodies whose sexualities are constructed by immorality (Mbembe, 2018).

Caravaca-Morera and Padilha (2018) believe that necropolitics applied on the LGBT body can be understood as a “social gear” that produces, in all spheres, practices that are managed through death, invisibility, and exclusion processes. The death policy authorized by the state is a “*condition for acceptability of making people die*” (Mbembe, 2018: 18), which acts on LGBT bodies beyond LGBTphobic discrimination. It endorses the reproduction of stigmas, school bullying, physical abuse, denial of city spaces, and even homicide. At this point, the state seems exempted from taking the necessary actions to protect these bodies when it is on the agenda because they are bodies that should not exist and they are lives that have strayed from the social norm and that need to remain precarious so that an imperialist logic, as Mbembe asserts, can remain.

By analyzing the death policy that acts on the bodies and sexualities understood as dissidents, it can be perceived how it remains influential and in motion in all social fields. Above all, it is visible that heteronormativity is a social construction that insists on being hegemonic with the ultimate aim of decimating specific populations. In this sense, it is extremely important to bring forth sexual diversity and denounce hegemonic practices that mortify LGBT bodies and other bodies considered not universal and thus breaks the silence against these totalitarian and immeasurable powers.

## Diffuse Violence and the Struggle for Recognition of Marginalized Individuals

From the postulated existence of a death policy aimed at dissident bodies, the LGBT population in this case, we can consider the possible consequences and the claims of individuals or social groups vulnerable to violence. In this sense, the notion of violence is developed and complexified due to the ways in which it can be expressed: symbolic, moral, physical, psychological, lethal, among many. The forms of social reaction to violence can vary due to the shared moral evaluation about the deviant act, who committed it and against whom this act was committed. As it is, there is scope for an interpretation produced and shared socially by individuals recognized and legitimized through their public behavior. However, we observe that the phenomenon of violence is configured as a structural factor of society. Therefore, how to identify and comprehend these dynamics between the death policy and the recognition of marginalized individuals?

We started from the premise of the idea of *deviance* to analyze how death policies can be operated against certain social groups. This idea comprehends social behaviors considered undesirable, denoting the structures of power relations that can legitimize arbitrariness against marginalized individuals or the social nonrecognition of their situation as a victim of such violence. Thus, we can refer to Goffman about social stigmas being also a kind of identity constructed, or attributed, by a public moral career, in which the stigmatized person is not considered entirely human (Goffman, 1978). These behaviors considered deviant are often surveilled, which can generate, at its limit, a process of collective criminalization – actually *a priori*, which may result in the selective suppression of the rights of marginalized groups.

To sociologically systematize the previous arguments, we will work on the concept of diffuse violence. This violence can be identified as political, social, gender, race, symbolic, ecological, among others (Tavares-dos-Santos, 2009: 83). According to sociologist José Vicente Tavares-dos-Santos (2009), the records of criminal rates of homicides and property crimes have intensified the feeling of insecurity, which can produce a type of diffuse social fear. Thus, diffuse violence shapes social relations and forms of sociability in contemporaneity, such as defensive behaviors. However, this feeling of widespread insecurity is more prominent in those who are most vulnerable to violence of all kinds, especially in urban spaces in large and medium-sized cities. Here, we observe the historical process of disorderly and accelerated urban development in Latin America, which includes Brazil, being spatially and socially heterogeneous and unequal. This had an effect on urban management, on types of segregation, and on the process of categorizing marginalized individuals by territorialization (Carman et al., 2013; Carrión, 2016).

Based on the concept of diffuse violence, Tavares dos Santos developed the sociology of conflictualities. This branch of sociology is based on the principle that social relations are conflicting by nature, thus considering conflictualities as being the driving force of the social field (Tavares-dos-Santos, 2009). The ideas of social complexity, the continuity, or discontinuity of the historicity of social processes and conflicts are the basis of the sociologist’s theory, which is used to analyze the political, cultural, and social changes in Brazil and Latin America. For Tavares dos Santos, the sociological reconstruction of the Brazilian social reality in contemporary times is a starting point for the analysis of rules and social conflicts. Globalization is perceived as a complexifying factor of social space, due to its capability of redefining values and rules, while diffuse violence emerges as a variable in this process.

Tavares dos Santos indicates how violence can have positive or negative connotations in Brazil. Diffuse violence can be deemed as a potency of social disintegration, debasing the concepts of citizenship and democracy in Brazil, and limiting social participation through the use of violence in the forms of injury, coercion and force. Social manifestations can be repressed violently if they collide with a certain social order. It directly affects the issue of criminalizing behaviors that *a priori* are social demand vindications that later generate conflicts. However, violence can be legitimized to maintain order and an immediate way to resolve social conflicts. It is evident then that punitive violence can considered positive for the dominant classes. This ambivalence of violence in Brazil, for Tavares dos Santos, showcases how democracy remains an unfinished process in this country.

Diffuse violence can be understood as a guide for defensive and vindictive behaviors. In Latin America, the processes of formal redemocratization after years of dictatorships opened up spaces for new actors in the institutional public sphere, denoting a multiplicity of demands and conflicts. In this sense, the quick and increasingly comprehensive changes of a globalized world produce subjectivities and subjections for the recognition of social groups. British criminalist Jock Young (2002) argues that these changes have highlighted the end of the consensual and functionalist world of the core countries of capitalism. Unquestionable values and certainties were deconstructed, especially after the cultural revolution of the 1960s, dynamizing individualism, diversity, and contesting structural models and social values. Thus, the criminalist claims that there is no fully inclusive society (Young, 2002: 16–18).

Young affirms that there is an exclusionary society – actually, an *exclusive society*, in his words – that produces areas of exclusivity, since the rise of individualism. The crisis of this apparently inclusive system took place during the era of controversy, denoted by plurality, debate, experimentation of disorder, and rebellions as well as by social frustrations of groups that have not been historically contemplated by the welfare state. Young says that this transition spanned the 1980s and 1990s, leading to an exclusion process in the perspective of the labor market and the social acceptance of certain groups. Therefore, the dialogue among Young and Tavares dos Santos is centered on diffuse violence since the criminalist addresses the loss of rights in the countries of the northern hemisphere, while the sociologist studies the demands for these rights in Latin America, especially in Brazil.

The gender dimension in diffuse violence is important for a better understanding of its manifestations in reality. The feelings of diffuse insecurity and fear among women were identified as ambivalent. If, on the one hand, it attests to the existence of vulnerability in relation to acts of violence, on the other hand, these feelings can produce group solidarity when it is recognized that these are not individual situations but collective within a power structure. Gender violence has become less tolerated, alluding to an emancipatory change for women. There have been several initiatives in the juridical field for the recognition of gender violence as a social problem, for instance, the feminicide law and the domestic violence law, *Maria da Penha,* in Brazil. However, the very existence of these laws demonstrates the persistence of violence against women, despite the legal norm and political advances.

There have been attempts to extend demands originally from the feminist movement to the LGBT population. Indeed, there can be intersections between them, but there are specificities as well. This extension worked in the implementation of the *Maria da Penha* Law, which regards aggressions in the domestic sphere, to LGBT couples as well as straight ones. However, other historical demands from the feminist movement may clash with demands from LGBT groups. The debate about the legalization of gestacional surrogacy is a delicate topic among homosexual couples and radical feminists, for example.

The scenario of diffuse violence denotes other types of vulnerabilities for the LGBT population. What is considered deviance can also be understood as a dissenting body for biological reasons, which can legitimize its physical elimination within a context of social and moral conservatism. Therefore, the uncertain world described by Young is aligned with the human incompleteness of the stigmatized presented by Goffman. However, exclusivity zones also compete as security areas for this group, based on spatial self-segregation, demonstrating the paradox of marginalization in a scenario of diffuse violence. In other words, certain urban spaces can be a safe haven, in which group solidarity can fuel demands about their right to exist before the state.

## Conclusion

This article sought to comprehend the notion of the city beyond universality, acknowledging the existence of death policies implemented by the aegis of hegemonic power. There are violent, hostile, and exclusionary processes regarding non-heteronormative bodies as a result of coercive impositions and mortifying praxis that aim to meet socially established norms and classifications. There is however hope as these bodies marked by sexual difference continue to exist on the public sphere.

For this reason, Benedetti (2005) expressed that bodies transform and reinvent themselves and experience different sensations, bringing forth the power to manifest their desires in a continuous resistance to the meanings posed by normative conceptions. We sought to reflect on the social relationships experienced by LGBT bodies, which, at the present juncture, still see themselves subjected to a colonial phantom. Therefore, it is from the body as an artifact for the construction of subjectivity that these fabrications were outlined as antithesis to hegemonic, moralizing, and universal standards.

Approaching the city and dissident bodies, we verified violence as a variable for producing territories and individuals in the urban space. In the text, we applied the concept of diffuse violence to understand how socially widespread fear and insecurity are produced. In Latin America, the objective criteria for measuring violence as a widespread catalyst for fear and insecurity are homicide rates, with the highest recurrence of this crime being recorded in cities. The concept of diffuse violence helped us analyze the vulnerability of the LGBT population and what are the possible mechanisms and strategies of defense. Territories can have different meanings in the urban space by the perspective of a cognitive mapping of public behaviors, demonstrating the ambiguity of feeling protected or threatened in the city.

Finally, from the articulation among the concepts of diffuse violence and necropolitics, we can elaborate some answers for those who exist in the city. First, the city as a place of encounter, surprise, and novelty can be an ambiguous statement, indicating negative and positive experiences. Second, it is possible to take violence into account as a guiding variable for public behavior for historically stigmatized social groups. These stigmas constitute a situation of vulnerability that helps us understand the splits between imagined and real violence and the nuances of this diffuse sense of insecurity and feeling of fear. Third, the gender dimension of diffuse violence conduces us to comprehend the social dynamics that legitimize or resist the elimination, both physical and symbolic, of the dissident body in the city.

Therefore, what was sought in this text was to problematize plurality within a hegemonic thought of universality of cities. We agree with Jock Young’s statement that there is no 100% inclusive society and, by applying the concept of diffuse violence, we were able to identify the vulnerability of historical individuals to necropolitics, for reasons of class, race, gender, among others. In this sense, we observe that the existence of LGBT bodies in the urban space is strongly outlined by resistance and persistence, with social solidarity and esteem toward these individuals varying in time and space.

## Data Availability

The raw data supporting the conclusions of this article will be made available by the authors, without undue reservation.

## Footnotes

1 Brazilian/Portuguese version. Original title from french: Le Droit à la Ville.

2 Translated from portuguese language.

3 Translated from portuguese language.

4 Translated from portuguese language.
